# The impact of anti-HBc positivity on clinical outcomes and treatment response in immune thrombocytopenia

**DOI:** 10.1007/s00508-025-02563-1

**Published:** 2025-08-11

**Authors:** Özlem Beyler, Cengiz Demir, Ali Doğan

**Affiliations:** 1 Department of Hematology, Gazi Yaşargil Training and Research Hospital, 21070 Diyarbakır, Kayapınar Turkey; 2 University of Health Sciences, Mekteb-i Tıbbiyye-i Şahane Complex, Tıbbiye Street No:38, Üsküdar, Istanbul, Turkey; 3 Department of Hematology, Van Yüzüncü Yıl University Faculty of Medicine, Van, Turkey

**Keywords:** Hepatitis B core antigensc, Autoimmune diseases, Platelet count, Immunologic factors, Treatment outcome

## Abstract

**Purpose:**

Immune thrombocytopenia (ITP) is an autoimmune disorder characterized by low platelet counts due to the destruction of platelets by autoantibodies. The presence of hepatitis B core antibodies (anti-HBc) is a marker of previous or occult hepatitis B virus (HBV) infection, which may influence the clinical course and treatment outcomes in ITP patients.

**Methods:**

This retrospective study investigated the impact of anti-HBc positivity on the clinical outcomes and response to treatment in ITP patients. A total of 116 patients with ITP were divided into 2 groups: those who were anti-HBc positive (*n* = 31) and those who were anti-HBc negative (*n* = 85). Clinical data, including platelet counts at diagnosis and during follow-up, response to corticosteroid treatment and remission rates were analyzed.

**Results:**

The results indicated that anti-HBc positive patients had significantly lower platelet counts at diagnosis (*p* = 0.009) and a longer hospital stay (*p* = 0.042). Additionally, these patients demonstrated lower complete response rates to initial corticosteroid treatment at 1 month (*p* = 0.001) and 6 months (*p* = 0.021) compared to anti-HBc negative patients; however, there were no significant differences in overall response or durable response rates between the groups at 12 months. The risk of relapse was higher in anti-HBc positive patients (*p* = 0.041).

**Conclusion:**

These findings suggest that anti-HBc positivity may be associated with a more severe disease course and reduced treatment efficacy in ITP, highlighting the importance of assessing hepatitis B serological markers when managing ITP patients.

## Introduction

Immune thrombocytopenia (ITP) is a hematological disorder characterized by the destruction of circulating platelets mediated by immunoglobulin G (IgG) autoantibodies, leading to thrombocytopenia, purpura and an increased risk of bleeding. The diagnosis of ITP is established through the exclusion of other potential causes of thrombocytopenia [1]. According to the American Society of Hematology, ITP is defined as a platelet count below 100,000/μL without associated abnormalities in leukocyte count or hemoglobin levels. Any deviations in these parameters typically necessitate further investigation to identify alternative etiologies [2].

Epidemiological studies suggest an annual incidence of ITP in adults ranging from 1 to 6 cases per 100,000 individuals [3–7]. Due to its chronic course in adults, the prevalence is considerably higher, with a U.S.-based study reporting approximately 12 cases per 100,000 individuals [6]. The ITP is classified into two forms: primary and secondary. Primary ITP accounts for approximately 80% of newly diagnosed adult cases and lacks an identifiable underlying cause. Secondary ITP, however, is associated with chronic infections, autoimmune diseases, malignancies, or exposure to certain medications [8–10]. Hepatitis B virus (HBV) infection remains one of the most common chronic viral infections worldwide, with varying prevalence across geographical regions and socioeconomic settings. Isolated anti-HBc positivity is a common serological profile in HBV endemic areas and is indicative of past HBV infection [11]. The frequency of isolated anti-HBc IgG positivity ranges from 0.1% to 20% globally and is reported as 3–5% in Turkey [12, 13]. Studies suggest that HBV infection may be linked to the pathogenesis of ITP. For instance, one study found the prevalence of ITP to be 6.35% in HBV-infected individuals [14]. Moreover, small-scale studies have reported that approximately 30.3% of ITP patients are anti-HBc positive [15]. Despite these observations, data on the prevalence of occult HBV infection in ITP patients and its impact on disease severity and treatment outcomes remain limited. Isolated anti-HBc positivity, as a marker of previous HBV exposure, may influence the clinical course and therapeutic response in ITP patients, but this area has not been thoroughly investigated.

This study aimed to evaluate the seroprevalence of anti-HBc positivity in a cohort of ITP patients. Additionally, we sought to explore the relationship between anti-HBc positivity and clinical parameters, such as platelet count and bleeding tendency, and assess the impact of anti-HBc status on treatment response in ITP.

## Material and method

This retrospective study aimed to evaluate the proportion of hepatitis B surface antigen (HBsAg)-negative and hepatitis B core antibody (anti-HBc)-positive individuals among patients diagnosed with ITP. Additionally, it examined the relationship between anti-HBc positivity, the severity of ITP, and treatment responses. The study was conducted in line with the Declaration of Helsinki and received ethics committee approval. As it was retrospective, patient consent was not required beyond ethics committee consent, as no additional tests were conducted beyond routine diagnostics. Medical records of ITP patients followed at the Gazi Yaşargil Training and Research Hospital and Van Yüzüncü Yıl University Faculty of Medicine Hematology Clinic between May 2019 and March 2024 were reviewed. Data included platelet counts at diagnosis and at 1, 6 and 12 months after treatment initiation, treatment protocols and demographic characteristics. A total of 116 patients were included, with data extracted from electronic medical records comprising complete blood counts, hepatitis B serological markers, basic biochemical tests, and treatment details. Hepatitis B markers (HBsAg, anti-HBs, HBeAg, anti-HBe, and anti-HBc IgG) were assessed using enzyme-linked immunosorbent assay (ELISA). Hepatitis B virus (HBV) DNA was quantified using reverse transcription-polymerase chain reaction (RT-PCR). Blood counts and biochemical parameters, including alanine aminotransferase (ALT), aspartate aminotransferase (AST), alkaline phosphatase (ALP), bilirubin, albumin and creatinine, were measured using automated analyzers. The patients were divided into two groups: group 1, which included 35 individuals who were HBsAg-negative and positive for both anti-HBc and anti-HBc IgG and group 2, which included 81 individuals who were HBsAg-negative and negative for anti-HBc IgG. The groups were compared in terms of demographic data (age, gender), laboratory findings (platelet count, hemoglobin, liver enzymes), treatment regimens (corticosteroids, immunoglobulin, other drugs), and disease outcomes (response (R), remission duration, relapse frequency). Exclusion criteria included malignancy, chronic liver disease, HIV or HCV infection, active HBV infection, or other comorbidities impacting ITP management. In our study, chronic liver disease was firstly excluded based on laboratory parameters such as normal liver function tests (AST, ALT, albumin, bilirubin), absence of clinical or biochemical findings suggestive of liver dysfunction. Secondly, normal spleen size (ruling out hypersplenism, a common cause of thrombocytopenia in advanced liver disease), normal liver parenchymal echogenicity and absence of ascites, portal vein abnormalities, or hepatosplenomegaly on abdominal ultrasonography. Initial treatment consisted of methylprednisolone (1 mg/kg per day), tapered upon achieving platelet counts ≥ 100 × 10^9^/L. Patients unresponsive to corticosteroids after 4 weeks received second-line treatment, including rituximab (375 mg/m^2^, 4 doses) or eltrombopag (50 mg/day, adjusted as needed). Anti-HBc-positive patients treated with rituximab received entecavir (0.5 mg) as antiviral prophylaxis. Primary endpoints included response rates and relapse frequency. A complete response (CR) was defined as a platelet count > 100 × 10^9^/L, while a durable response required a platelet count ≥ 30 × 10^9^/L, with at least a twofold increase from baseline at 6 months. A relapse was defined as a platelet count < 30 × 10^9^/L or new bleeding episodes after a prior response.

Statistical analyses were performed using SPSS 21.0 (IBM Corporation, Armonk, New York, United States). Descriptive statistics were expressed as mean, standard deviation, minimum and maximum values for continuous variables and frequencies for categorical variables. Independent t‑tests or Mann-Whitney U tests were used for group comparisons, depending on normality assumptions. Correlations were assessed using Pearson or Spearman coefficients and χ^2^-tests were used to evaluate categorical data, with significance set at *p* < 0.05.

## Results

Of the 116 patients included in the study 26.7% (*n* = 31) were anti-HBc IgG positive and 73.2% (*n* = 85) were anti-HBc IgG negative. When the gender distribution between the groups was analyzed, the proportion of males was higher in the anti-HBc IgG positive group (61.2%) compared to the negative group (37.6%) (*p* = 0.001). The mean ages were 47.0 ± 8.0 years and 41.0 ± 9.0 years, respectively. The mean age of the anti-HBc IgG positive group was statistically significantly higher than the negative group (*p* = 0.001). The mean age of the general cohort was calculated as 44.0 ± 10.0 years (Table 1). The mean duration of hospital stay was 8.8 days in anti-HBc IgG positive patients and 7.5 days in anti-HBc IgG negative patients. This difference between the two groups was statistically significant (*p* = 0.042; Table 1). At the time of diagnosis, the mean platelet count was found to be significantly lower in the anti-HBc IgG positive group compared to the negative group (*p* = 0.009). On the other hand, hemoglobin levels were lower in the negative group compared to the positive group (*p* = 0.032). Leukocyte counts, creatinine, AST and ALT levels did not show a statistically significant difference between the two groups (*p* = 0.123; *p* = 0.081; *p* = 0.058 and *p* = 0.727, respectively). These findings are presented in detail in Table 2. Data evaluating the efficacy of initial methylprednisolone treatment in anti-HBc IgG positive and anti-HBc IgG negative patients were obtained from the patient files, 22 patients who did not respond to treatment with corticosteroids were treated with 4 doses of rituximab 375 mg/m^2^. The number of patients who were treated with corticosteroids alone was 32. Hbc IgG was positive in 8 of the patients receiving corticosteroids alone. Hbc IgG positivity was found in 12 of the 22 patients receiving rituximab. Results of cross-tabulation analysis indicate that among the 22 patients who received rituximab, 11 (50%) were anti-HBc IgG positive, while among the 94 patients who did not receive rituximab, only 13 (13.8%) were anti-HBc IgG positive. The expected counts suggest that more patients with anti-HBc IgG positivity than expected received rituximab. The initial complete response rate of the anti-HBc IgG positive group was lower than that of the anti-HBc IgG negative group (48.3% vs 61.1%, *p* = 0.001). Although overall response rates were higher in the anti-HBc IgG-negative group, no statistical difference was observed in overall responses (including CR and R) between the anti-HBc IgG positive and negative groups (70.9% vs, 82.3%, *P* = 0.086; Table 3). In terms of durable response at 6 months, durable response was observed at similar rates in both anti-HBc IgG positive and negative patients (41.9% vs. 48.2%, *p* = 0.438); however, as in the initial complete remission rates, the complete remission rate was significantly lower in the anti-HBc IgG positive group compared to the negative group at 6 months (25.8% vs. 42.3%, *p* = 0.021). These results are presented in Table 3. When treatment responses were evaluated at the 12th month of the study, no significant difference was found between anti-HBc IgG positive and negative patients in terms of durable response rates (35.4% vs. 41.1%, *p* = 0.247); however, the complete remission rate was significantly lower in the anti-HBc IgG positive group compared to the negative group at 12 months (19.3% vs. 27%, *p* = 0.041). These findings are presented in detail in Table 3. The risk of recurrence was analyzed in all first responder patients. Compared with anti-HBc IgG negative patients, the risk of relapse was significantly higher in anti-HBc IgG positive patients. At the end of 12 months, complete responses were maintained in 19.3% of anti-HBc IgG positive patients and 27% of anti-HBc IgG negative patients (*p* = 0.041).

**Table 1:** Demographic and clinical features of patients with and without anti-HBc IgG positivity

	Anti-HBc IgG positive (*n* = 31)	Anti-HBc IgG negative (*n* = 85)	*p*-value
Age (years, mean ± SD)	47.0 ± 8.0	41.0 ± 9.0	*0.001*
Male, *n* (%)	19 (61.2)	32 (37.6)	*0.001*
Female, *n* (%)	12 (38.7)	53 (62.3)	*0.002*
Duration of hospital stay (days, mean ± SD)	8.8 ± 1.2 (6–10)	7.5 ± 1.0 (5–9)	*0.025*

**Table 2:** Mean laboratory values of both groups

	Anti-HBc IgG positive (*n* = 31)	Anti-HBc IgG negative (*n* = 85)	*p*-value
WBC (× 10^9^/L, mean ± SD)	5.8 ± 0.8	6.3 ± 0.9	0,123
Hemoglobin (g/dL, Mean ± SD)	14.7 ± 1.2	13.5 ± 1.4	*0,032*
Platelets (× 10^9^/L, mean ± SD)	7.2 ± 4.5	10.5 ± 5.3	0.0095
Creatinine, (mg/dL, mean ± SD)	0.72 ± 0.11	0.69 ± 0.15	0,081
AST (IU/L, mean ± SD)	25.57 ± 4.63	23.99 ± 4.15	0,058
ALT (IU/L, mean ± SD)	18.78 ± 6.23	19.16 ± 5.31	0,727

*WBC* white blood cell count, *AST* aspartate aminotransferase, *ALT* alanine aminotransferase, *SD* standard deviation, *HBc* hepatitis B core, *IgG* immunoglobulin G. Italicized numbers indicate values with statistical significance (*p* 0.05).

**Table 3:** Initial response and sustained response in the overall cohort

	Anti-HBc IgG positive (*n* = 31)	Anti-HBc IgG negative (*n* = 85)	*p*-value
Initial response			
OR, *n* (%)	22 (70.9)	70 (82.3)	0.086
CR, *n* (%)	15 (48.3)	52 (61.1)	*0.001*
Sustained response			
*Month 6*			
Sustained response, *n* (%)	13 (41.9)	41 (48.2)	0.438
Sustained CR, *n* (%)	8 (25.8)	36 (42.3)	*0.021*
*Month 12*			
Sustained response, *n* (%)	11 (35.4)	35 (41.1)	0.247
Sustained CR, *n* (%)	6 (19.3)	23 (27.0)	*0.041*

*Overall response (OR)* was defined as CR or R.

*Sustained response (SR): *a response (CR and R) lasting for at least 6 months without any ITP-specific treatments

*Complete response (CR):* platelet count ≥ 100,10^9^/L and absence of bleeding

*Response (R):* platelet count ≥ 30 × 109/l and at least a twofold increase of the baseline platelet count and absence of bleeding

*No response (NR):* platelet count < 30 × 109/l or a < twofold increase of the baseline platelet count or bleeding.

## Discussion

The ITP is an acquired thrombocytopenia syndrome characterized mainly by autoantibody-mediated platelet destruction and megakaryopoiesis dysfunction. Viral infections, especially hepatitis B and C viruses, are considered to be an important trigger in the development of ITP. Immune dysregulation caused by these viruses may predispose to ITP by increasing autoantibody production and by direct or indirect effects on megakaryopoiesis. Studies have reported that the incidence of ITP in patients with chronic HCV and HBV infections is 11.86% and 6.35%, respectively [14]. HBV infection is recognized as a global health problem. According to 2018 data, approximately 3.5% of the world population are chronically infected with HBV [16, 17]. The highest HBV prevalence has been reported in China [18]. The clinical course of HBV infection is a complex result of the interaction between viral replication and the host immune system [19]. Occult HBV infection is characterized by serum HBsAg negativity and anti-HBc positivity with detectable or undetectable anti-HBs and HBV DNA is usually undetectable [20]. There is only one study in the literature on the effect of the presence of occult HBV infection on disease severity and treatment response in ITP [21]. In studies, the prevalence of isolated anti-HBc IgG varies between 0.1% and 20% in different populations [22]. In the study by Wang et al., the rate of anti-HBc positivity in ITP patients was reported to be 51% [21]. This rate is higher than the general prevalence among people living in China. They suggested that the reason for the higher anti-HBc IgG positivity in the ITP patient group compared to the normal population may be the age range of the study population. The patients included in this study were aged 13–82 years, whereas individuals in the previous epidemiological study were aged 1–59 years [23]. Similarly, we found a higher rate of anti-HBc IgG positivity in our ITP patient group compared to the normal population. The prevalence of isolated anti-HBc IgG is relatively high in Turkey, we found this rate to be 26.73% in our study [24]. The rate we observed is lower than the rate found in the study by Wang et al. but the frequency of 30.3% reported by Pivetti et al. in ITP patients is similar to the findings of our study [15]. Considering these rates, it is clear that the prevalence of anti-HBc IgG is higher in ITP patients compared to the general population. A possible explanation for this may be that the probability of exposure to HBV increases with age. In both our study and the study by Wang et al. the mean age in the anti-HBc IgG positive group was significantly higher than in the negative group [21]. While some studies confirmed female predominance in young adults, others did not. In most studies, there is a similar incidence in men over 60 years of age compared to women [3, 6, 25]. In the study by Wang et al. similar to the general ITP population, female gender was significantly more prevalent than male gender in both anti-HBc IgG positive and negative groups [21]; however, in our study, females were more prevalent in the anti-HBc IgG negative group, whereas there was male dominance in the anti-HBc IgG positive group, contrary to the findings of Wang et al. This may have been influenced by the higher mean age of the anti-HBc IgG positive group compared to the negative group. In the study by Wang et al., baseline platelet counts were compared between anti-HBc IgG positive and negative ITP patients and platelet counts were found to be lower in the positive group. Similarly, in our study, platelet counts were found to be lower in the anti-HBc IgG positive ITP group compared to the negative group. This has been interpreted as a possible alteration in the immune response, although the viral load could not be detected [21]. Anemia or leucopenia is not expected in ITP [26]. In our study, mean leukocyte and hemoglobin levels at diagnosis were within normal ranges and were similar in both groups; however, the mean hemoglobin level was lower in the anti-HBc IgG negative group compared to the positive group, although not in the anemic range. This was thought to be due to the fact that most of the patients in the anti-HBc IgG negative group were women and iron deficiency was present in most of them. As in a previous study [21] the mean hospital stay of anti-HBc IgG positive patients was longer than that of the negative group in our study. This finding suggests that anti-HBc IgG positivity may adversely affect the course of the disease in ITP patients, leading to a longer and more severe disease course and potentially contributing to resistance to treatment. This emphasizes the importance of evaluating hepatitis B serological markers in ITP patients and taking this factor into consideration when determining treatment strategies. In our study, we could not compare the bleeding scores between the two groups due to insufficient data; however, Wang et al. reported that the anti-HBc positive group had higher bleeding scores compared to the negative group [21]. We treat most newly diagnosed ITP patients with platelet counts < 20 × 10^9^/L, even in the absence of bleeding symptoms. Typical initial treatment involves administration of a glucocorticoid (dexamethasone or methylprednisolone) [27]. Long-term complete remission with glucocorticoids has been reported in approximately 20% of individuals based on uncontrolled studies. A 2016 meta-analysis of 9 randomized trials involving 1138 newly diagnosed ITP patients treated with 1 mg/kg oral prednisone for 2–4 weeks found that the rate of achieving a platelet count > 30,000/microL at 2 weeks was 59% [28]. In our study, we administered methylprednisolone 1 mg/kg as initial treatment to both anti-HBc IgG positive and negative ITP patients. When the responses to initial treatment were compared between the two groups, the overall response rate was higher in the anti-HBc IgG negative group compared to the positive group, but this difference was not statistically significant. This finding is consistent with the results of Wang et al. who found no difference in overall responses to initial treatment between anti-HBc positive and negative groups [21]; however, we found that the complete response rate to initial treatment was significantly lower in the anti-HBc IgG positive group compared to the negative group; this result is similar to that reported by Wang et al. [21]. This suggests that anti-HBc IgG positivity may affect treatment response and may adversely affect deep responses due to its effects on immune status. In a study involving HBsAg-negative and anti-HBc IgG-positive patients receiving corticosteroids, the HBsAg seroreversion rate was 1.8% (1-year incidence) [29]. This suggests that corticosteroid use in patients with a history of HBV infection (anti-HBc IgG positive) may increase the risk of HBV reactivation. In our study, HBV reactivation was not detected in the anti-HBc IgG positive patient group. Recurrence was higher in anti-HBc IgG positive patients compared to negative patients. This contradicts the findings of Wang et al. who reported no difference in the risk of recurrence between the two groups [21]. In conclusion, both our study and other studies in the literature show that anti-HBc IgG positivity is significantly higher in ITP patients compared to the general population. Anti-HBc positivity is associated with more severe clinical outcomes, such as lower platelet counts, higher bleeding risk and prolonged hospitalization, and may adversely affect prognosis and treatment response in ITP patients. Although our study focused on patients with isolated anti-HBc positivity, the inclusion of patients with active chronic hepatitis B infection (CHB) may provide additional information. Future studies should investigate corticosteroid response in ITP patients with CHB as CHB may also be associated with a different immunological status and potentially an altered response to immunosuppressive therapy. Therefore, the response to immunosuppressive therapies such as corticosteroids may differ from the response in patients with isolated anti-HBc positivity. Comparative analyses between CHB-ITP and occult HBV-ITP cohorts may provide potential insights into disease behavior, treatment response, and specific therapeutic approaches in these subgroups.

Moreover, this study has several limitations. First, its retrospective design limits the ability to make definitive causal inferences. Second, there were significant differences in baseline characteristics such as age and sex between the anti-HBc positive and negative groups, which may have influenced clinical outcomes and treatment responses. Third, although all patients were evaluated by clinical examination, routine laboratory tests, and abdominal ultrasonography, these methods cannot completely exclude the presence of compensated advanced chronic liver disease. Future prospective studies should consider including noninvasive techniques such as transient elastography to more accurately assess liver fibrosis in ITP patients with prior HBV exposure.
